# Proximal humerus fracture and acromioclavicular joint dislocation

**DOI:** 10.1515/iss-2023-0049

**Published:** 2024-04-11

**Authors:** Maren Bieling, Alexander Ellwein, Helmut Lill, Stephan Sehmisch, Freya Margaretha Reeh

**Affiliations:** Klinik für Orthopädie und Unfallchirurgie, DIAKOVERE Friederikenstift 246722 , Hannover, Germany; Klinik für Unfallchirurgie, Medizinische Hochschule Hannover, Hannover, Germany

**Keywords:** proximal humerus fracture, joint-preserving locking plate fixation, reverse total shoulder arthroplasty, acromioclavicular joint instability, arthroscopic assisted Endobutton systems, horizontal instability

## Abstract

Proximal humerus fractures and injuries to the acromioclavicular joint are among the most common traumatic diseases of the upper extremity. Fractures of the proximal humerus occur most frequently in older people and are an indicator fracture of osteoporosis. While a large proportion of only slightly displaced fractures can be treated non-operatively, more complex fractures require surgical treatment. The choice of optimal treatment and the decision between joint-preserving surgery by means of osteosynthesis or endoprosthetic treatment is often a difficult decision in which both fracture morphology factors and individual factors should be taken into account. If endoprosthetic treatment is indicated, satisfactory long-term functional and clinical results have been achieved with a reverse shoulder arthroplasty. Injuries to the acromioclavicular joint occur primarily in young, athletic individuals. The common classification according to Rockwood divides the injury into 6 degrees of severity depending on the dislocation. This classification forms the basis for the decision on non-operative or surgical treatment. The indication for surgical treatment for higher-grade injuries is the subject of controversial debate in the latest literature. In chronic injuries, an autologous tendon transplant is also performed. Whereas in the past, treatment was often carried out using a hook plate, which was associated with complications, the gold standard today is minimally invasive treatment using Endobutton systems. This review provides an overview of the two injury patterns and discusses the various treatment options.

## Introduction: proximal humerus fracture

### Epidemiology and etiology

With an incidence of 105–342 per 100,000 [1], the fracture of the proximal humerus presents one of the most common injuries, especially in elderly people [2], and thus accounts for 4–5 % of all fractures in adults [3], [4], [5]. Since the risk of suffering a proximal humerus fracture rises with increasing age due to a reduced osteoporotic bone quality, it constitutes one of the so-called indicator fractures for osteoporosis [6] and its incidence is expected to three times more over the next three decades [7, 8]. Due to their higher incidence of osteoporosis, women, in particular, bear an increased risk of suffering these fractures [9]. In younger patients, fractures of the proximal humerus occur following high energetic trauma, such as motor vehicle crashes (MVCs) [5] or sports accidents – in general leading to fractures with highly complex morphologies as well as possible concomitant soft tissue and/or neurovascular injuries.

For the majority of the proximal humerus fractures being minimally displaced non-operatively treatment offers a reasonable treatment option [7, 9]. However, controversy still remains as for the optimal care of displaced fractures with a variety of treatment options (e.g., open reduction internal fixation with a locking plate, intramedullary nail osteosynthesis or arthroplasty) with an increasing interest in surgical intervention to anatomically stabilize the fracture. In contrast, even in the case of displaced fractures, some experts choose a non-operative therapy concept [9], [10], [11], [12]. Consequently, optimized implant technology for fracture fixation has constantly been developed, especially with respect to the elderly patient cohort [3, 11]. Due to a limited number of level I and II studies, there have, nevertheless, not been any validated, evidence-based recommendations so far regarding the ideal treatment of displaced proximal humerus fractures [10], [11], [12].

### Diagnostics

With respect to the diagnostics of proximal humerus fractures, a targeted, well-structured anamnesis is performed focusing on the type of trauma, the current complaints as well as on patient-individual criteria (e.g., age, medical history in terms of comorbidities, individual demands). The patient usually keeps the respective injured upper extremity in a protective posture with an internally rotated and adducted arm. Because of the acute high level of pain, the quantification of the range of motion as well as the clinical examination of the rotator cuff muscles are habitually not possible to be carried out. Owing to its anatomical course of passing through the lateral axial gap and then moving from dorsal around the collum chirurgicum of the proximal humerus, concomitant lesions of the axillary nerve are possible to arise – resulting in sensory dysfunctions in the nerve’s autonomous innervation area, i.e., the lateral area of the shoulder. Irrespective of the type of treatment (non-operatively vs. surgical), partial and severe complete lesions of the axillary nerve, respectively, were found in 32 % of patients in a study by Fjalestad et al. [10] via the use of electromyography of the deltoid muscle with an increasing incidence in dislocation fractures [13]. In case of dislocation or severely dislocated proximal humerus fractures with verifiable neurological deficits, usually due to a direct compression of the nerve or the brachial plexus, an immediate surgical decompression is required via reposition of the fracture and internal stabilization in order to prevent further and persistent deficits [13, 14].

Standard radiological diagnosis to exclude fracture or glenohumeral dislocation includes native radiography in at least 2 planes (true a.p., Y-image, axial). Since patients following an acute trauma often do not tolerate a shoulder abduction of 60°–90° as required for an axial radiographic imaging of the shoulder, the Y-plane can be used instead. With a fracture of the proximal humerus being radiographically diagnosed, a succeeding computer-tomographic imaging with a three-dimensional reconstruction is mandatory to further analyze the morphology of the proximal humerus fracture (e.g., number and size of fragments, head-split component, dislocation fractures, rupture of the posteromedial periosteum (*medial hinge*), angulation of the humeral head >45°) as well as to generate first prognostically and therapeutically relevant information.

Since the adequate choice of primary treatment of the fracture of the proximal humerus is based on a meticulous analysis of its morphology with the help of suitable classification systems and the presence of possible, especially soft tissue, concomitant injuries as well as on individual patient-specific factors, the thorough diagnostics represents the most crucial basis within the course of the longtime treatment of a fracture of the proximal humerus.

### Classification

Based on the above-mentioned importance of a correct and thorough assessment of the complex morphology of proximal humerus fractures, the need for a practically useful classification of such fractures becomes particularly evident in order to enable a profound treatment decision making [15]. Apart from revealing a high inter- and intraobserver reliability, the aim of these classification systems is to provide prognostically and therapeutically relevant information as well as to prove applicable in everyday clinical practice. With regard to proximal humerus fractures, different classification systems have been developed, primarily basing on pathomorphological criteria, particularly fracture localization and fragment quantification [16]. Most of the currently available classifications base on the observations made by Codman et al. [17], proclaiming that a fracture of the proximal humerus results in four major fracture segments, in detail the humeral head superior to the anatomical neck, the greater tuberosity, the lesser tuberosity and the humeral shaft [13, 18].

The classification described by Resch et al. [16] is the most clinically relevant classification. Apart from focusing on the degree of the dislocation of the fracture, the angulation of the head fragment in relation to the humeral shaft (valgus-/varus malalignment) is furthermore taken into consideration, thus providing prognostically and therapeutically relevant information [19].

Despite the constant attempt to develop an ideal classification for proximal humerus fractures that fulfills the criteria of being completely exhaustive, clinically applicable as well as of providing prognostically and therapeutically relevant information, so far there has not been any classification thoroughly meeting these criteria. In order to nonetheless allow a profound treatment decision making, certain aspects regarding the morphology of the proximal humerus fractures have been identified to be particularly relevant (Table 1).

**Table 1:** Key aspects of the morphology of proximal humerus fractures.

– Number of fracture segments (i.e., 2- to 4-part fractures).
– Tilting angulation of the humeral head to the shaft (i.e., varus or valgus malalignment).
– Distracted vs. impacted fracture morphology.
– Integrity of the posteromedial periosteum (*medial hinge*).
– Length of the posteromedial fracture pick.
– Presence of a comminution zone.
– Presence of a head-split component and/or a dislocation fracture.
– Extent of the tuberosities’ dislocation.

### Therapy

Subsequent to a thorough diagnostics, focusing on the patient’s age, comorbidities, concomitant injuries, patient-individual entitlement as well as on the morphology of the proximal humerus fracture with particular respect to the aforementioned key aspects, all these pieces of information need to be assessed. As for the therapy of proximal humerus fractures, there are either non-operatively or surgical treatment options, elaborated on in the following.

### Non-operative treatment

The non-operatively treatment of proximal humerus fractures is an established and widespread procedure to treat stable, non- or minimally displaced proximal humerus fractures. With respect to indication criteria of non-operatively therapies, the current literature recommends non-operative treatment for fractures with a tilting angulation of <20°, a dislocation of <5 mm or a dislocation of the tuberosities of <2 mm [13, 15, 20]. Apart from this, prognostic predictors of an avascular humeral head necrosis and/or of a pseudarthrosis formation, both concerning the osseous healing of the fracture, are to be taken into account. In addition, patient-individual factors (e.g., age, general condition, surgical and anesthetic risk, comorbidities, functional requirement and entitlement, concomitant injuries) have to be also taken into consideration in the process of the decision making for a non-operatively treatment, in particular in the case of minimally displaced fractures [13, 20]. In contrast to the surgical treatment, the non-operative therapy offers the advantage of sparing neurovascular structures, therefore minimizes the potential risk of a further devascularization of the humeral head, and, in general, circumvents possible complications associated with surgical interventions [12, 13, 21]. Despite these evident advantages, instable and severely dislocated proximal humerus fractures as well as dislocation fractures or those with a head-split-component, however, represent contraindications to a non-operatively treatment. Pathologic fractures, open soft tissue injuries as well as closed irreducible shaft dislocations of >50 % account as further contraindications [13, 20].

During the first week succeeding the trauma, the non-operatively treatment consists of a temporary immobilization of the respective arm in a shoulder-arm-sling bandage or another type of immobilization orthoses. From the beginning onwards, the elbow and the wrist of the respective upper extremity can, nevertheless, be actively mobilized by the patient. Especially during the first weeks, it is important to support the passive and later on the active mobilization of the respective shoulder by means of an adequate analgetic therapy. Manual lymphatic drainage and cryotherapy should additionally be applied. During the second week, the patient is cautiously starting with pendulum exercises. At the beginning of the third week after the initial trauma, the physiotherapeutic treatment of the affected shoulder includes a functional mobilization with a free passive range of motion and first actively-assisted exercises without any load [13, 20]. At this point of the treatment, the shoulder-arm-sling is worn only intermittently. Throughout the following weeks, the physiotherapeutic treatment needs to be continued in order to gradually increase the passive as well as the active initial range of motion of the respective shoulder. As for therapy control, a first radiographic follow-up examination is to be done after 7–10 days. In case no secondary dislocation of the fracture can be divulged, the non-operatively treatment will be continued and further radiographic controls should be carried out after 3 and 6 weeks. In case a secondary dislocation of the fracture is, however, radiographically detected, possibly indicating an instable fracture morphology, a surgical treatment option should be re-considered [15]. In case the radiographic imaging after 6 weeks reveals a proper increase with regard to the osseous healing of the fracture without any signs of dislocation, the gradual increase in physical load will be recommended [15].

### Surgical treatment

With respect to the surgical treatment of a proximal humerus fracture, there is a great spectrum of treatment options – either joint-preservingly with an osteosynthesis or endoprosthetically with the implantation of an arthroplasty. In general, the joint-preserving osteosynthesis is especially favored for younger patients, whereas in older patients with among others preexisting degenerative lesions of the rotator cuff muscles, the implantation of a reverse total shoulder arthroplasty has been established as a primary intervention in the treatment of a proximal humerus fracture. Nevertheless, there are now some authors who select the therapeutic strategy regardless of patient age.

In line with their aforementioned particularities, sole fractures of the greater tuberosity with any or minimal dislocation can exclusively be addressed arthroscopically with a screw osteosynthesis or with an arthroscopically-based *packing* as performed in suture-bridging-technique. The latter represents a two-rowed anchor placement with interposed suture bridges leading to a two-dimensional fixation [15, 20, 22, 23].

With focus on the indication for a surgical treatment, there are absolute indications such as open fractures, dislocation fractures and fractures with a head-split component as well as concomitant neurovascular injuries. Complex and dislocated 4-part fractures as well as fractures with a greater extent of dislocation (>5 mm dislocation, >20° tilting angulation) are considered as relative indications for a surgical treatment. In these cases and in line with the above-mentioned, the individually different functional entitlement of the patient (e.g., activities of daily living, leisure activities, sport, work) is of crucial importance for the final treatment decision making [15, 20].

### Open reduction and locking plate fixation

**Figure 1:**
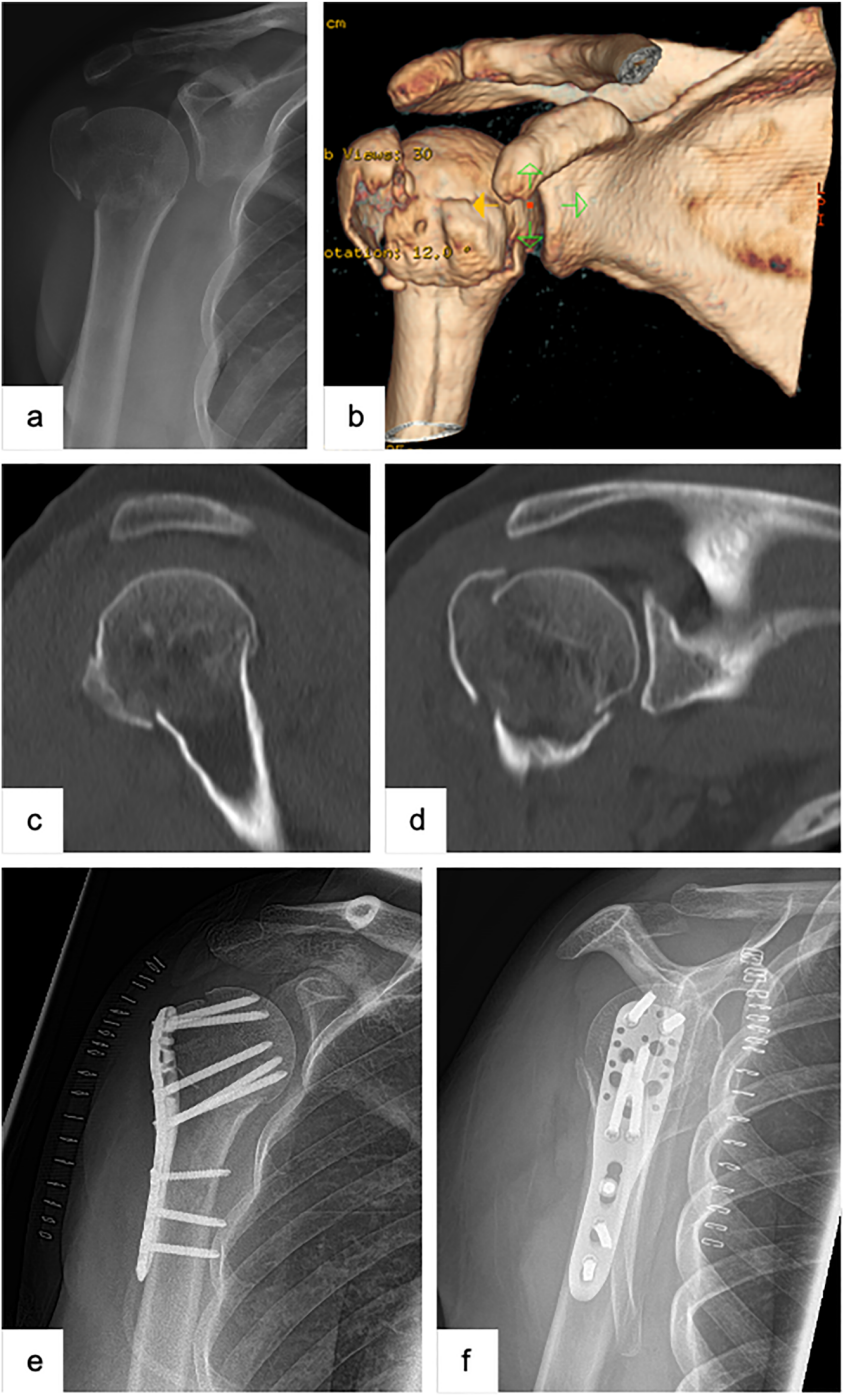
Female patient (53 y), right shoulder, valgus impacted, dislocated 3-part proximal humerus fracture (preoperative X-ray and (3D-) CT-scan: a – a.p., b – 3D-CT-reconstruction, c – sagittal, d – coronal), open reduction and internal fixation with a locking plate osteosynthesis (postoperative X-ray: e – a.p., f – Y-plane).

Dependent on the specific fracture morphology, the open reduction and Locking plate fixation (Figure 1) represents the main joint-preserving treatment option. Its aim is to achieve an anatomic reconstruction with a sufficiently medial support, thus leading to good functional results [15, 24], [25], [26].

The surgery is carried out by using the deltopectoral approach. The latter is an anatomic approach profiting from the physiologic muscle gap between the pectoralis major and the deltoid muscle with a minimized risk of damaging the axillary nerve. Furthermore, the deltopectoral approach allows to address all fracture morphologies (e.g., with a fractured lesser tuberosity) and possible concomitant injuries, such as fractures of the glenoid, as well as enables revision surgeries using the same approach.

In order to allow a suitable control over the tuberosities and its fragments, respectively, aiding the reduction of the fracture, both the greater and the lesser tuberosities are reinforced with a strong non-absorbable suture to later on use the latter as a supportive tension band. Further proceedings are dependent on the fracture’s morphology. In valgus-impacted fractures, for instance, the humeral head, first of all, needs to be mobilized into a more varus, non-impacted position before the shielding ring of the tuberosities can be closed. In case the fracture reveals a varus malpositioning, the humeral head first of all needs to be elevated to reposition the region of the medial calcar (i.e., humeral neck). The so-called calcar screws rising from caudo-lateral to cranio-medial should always be applied, in particular in fractures with a varus dislocation and therefore the need for a stabilizing medial support. In case the tendon of the long biceps is affected by the fracture’s line, by for instance involving the intertubercular sulcus, and in order to prevent a in particular ventrally localized postoperative pain, a tenodesis of the long biceps tendon should be carried out [15].

An immobilization of the respective shoulder is exclusively indicated initially to reduce the acute postoperative pain. In the following, the follow-up treatment includes a physiotherapeutic mobilization with no restrictions regarding the range of motion but without any load on the respective shoulder for a period of 6 weeks.

Despite the continuous development and the aim to improve implant design as well as surgical techniques, the osteosynthetic treatment of proximal humerus fractures is still associated with a high complication rate of 12.6 % [15, 20], among others leading to primary or secondary screw dislocation, i.e., a *cut out*, with a loss of reduction, infection, avascular necrosis of the humeral head, pseudarthrosis as well as other kinds of secondary fracture sequelae [3, 15, 20, 27]. In this context, the above-mentioned integrity of the *medial hinge* and the length of the metaphyseal extension of the humeral head fragment both represent prognostically relevant aspects. Hertel et al. [28], for instance, demonstrated that a combination of fractures at the collum anatomicum with a dislocation at the *medial hinge* of >5 mm and a metaphyseal extension of the humeral head fragment of <8 mm is linked to an increased risk of an avascular head necrosis. The among others age-dependently reduced bone quality, in particular of the humeral head, is another prognostically relevant aspect since it has been associated with an increased risk for implant failure due to a secondary loss of reduction. The high complication rate can, however, be reduced with a consecutively ameliorated clinical outcome by a meticulous analysis of the fracture’s morphology and correspondingly by a correct treatment decision making with particular focus on the question, when a joint-preserving treatment with an anatomic reduction and among others with a metaphyseal support, is indicated.

Apart from the use of calcar screws as well as of polyaxial ones which ameliorate the stable fixation thanks to inserting the latter into the dorso-medial part of the humeral head [15], the biomechanical stability of the plate osteosynthesis can, furthermore, be significantly increased by cement augmentation of the screws [15, 29, 30] or by using bone grafts (i.e., fibular grafts, femoral head, iliac crest chips) [31].

**Figure 2:**
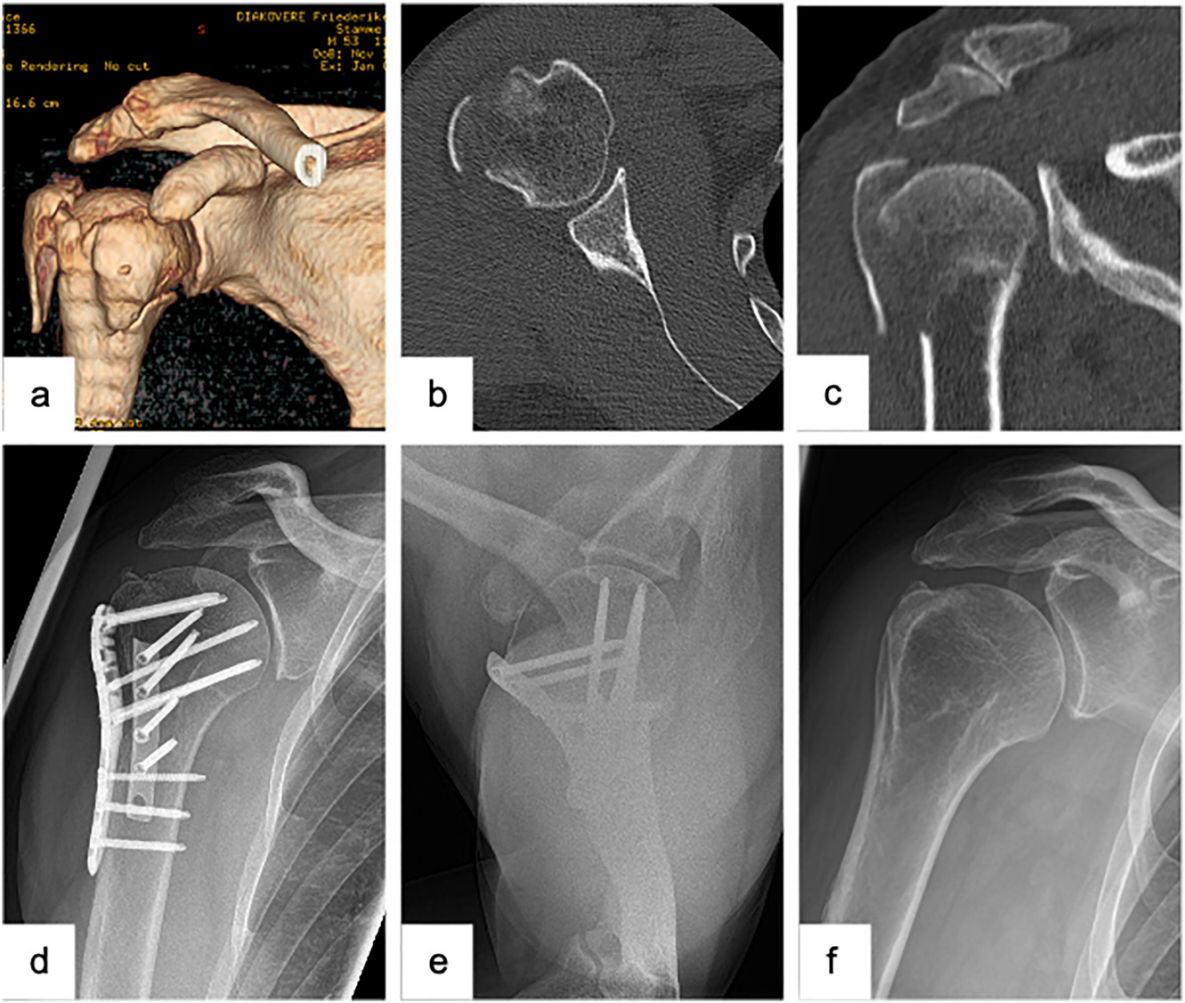
Male patient (53 y), right shoulder, valgus impacted 4-part proximal humerus fracture (preoperative (3D-) CT-scan: a – 3D-CT-reconstruction, b – axial, c – coronal), open reduction and internal fixation with a laterally positioned locking plate osteosynthesis and a second, supportive, ventrally positioned plate to stabilize the lesser tuberosity (postoperative X-ray: d – a.p., e − Y-plane), complete removal of the double plate osteosynthesis and arthroscopically assisted arthrolysis (postoperative X-ray: f – a.p.).

Another option to increase the stability of the reduction of complex fractures is the appliance of an additive support via a second, ventrally positioned plate (Figure 2). Apart from fractures with a reduced bone density, the latter is in particular crucial to highly instable fractures (e.g., fractures with a severely dislocated greater tuberosity, varus dislocated fractures with an insufficient medial support, fractures with a metaphyseal comminution zone). Both biomechanical and clinical results have so far shown promising results with respect to the use of an additive second plate [32].

### Intramedullary nail osteosynthesis

In addition to the Locking plate fixation, the intramedullary nail osteosynthesis accounts for another joint-preserving treatment option [15].

In contrast to the plate osteosynthesis, the deltoid-splitting approach is the one to be applied for the intramedullary nail osteosynthesis. In line with the aforementioned reinforcing of the tendons of the rotator cuff muscles in order to reduce the dislocated fragments of the tuberosities, non-absorbable sutures should be applied in an analogous manner. Due to the intramedullary nail’s limitations as for the treatment of complex multifragmentary fractures, the risk to injure the supraspinatus tendon and to cause a secondary subacromial impingement syndrome, intramedullary nail osteosynthesis is, therefore, preferably used for the treatment of 2- or 3-part proximal humerus fractures with meta- or diaphyseal involvement and without significant involvement of the humeral head and dislocation of the tuberosities [15, 20, 26].

### Arthroplasty treatment

Since the arthroplasty treatment has been divulged to lead to results inferior to those of an anatomic joint-preserving reconstruction, the indication to arthroplasty treatment of proximal humerus fractures has to be taken cautiously [15, 26]. However, as the clinical outcome after a secondary implantation of a shoulder arthroplasty succeeding a failed primary joint-preserving osteosynthetic treatment is worse than after a primary implantation of a prosthesis as a treatment for a proximal humerus fracture [33], the primary indication for a joint-preserving treatment of the proximal humerus vs. the primary implantation of a shoulder arthroplasty is one of the most crucial and sustainably relevant decisions within the course of the longtime treatment of a proximal humerus fracture and therefore needs to be meticulously made by taking all essential criteria into consideration (Table 2).

**Table 2:** Indication criteria for considering endoprosthetic treatment.

– Fractures with an impression of the humeral head (>40 % of the articular surface).
– Missing or short length of the posteromedial fracture pick.
– Fractures with a head-split component.
– Missing and non-reconstructable medial support of the calotte.
– Small cupped fragment of the calotte.
– Rupture of the posteromedial periosteum (*medial hinge*).
– Dislocation fractures persisting in luxated position for >48 h.
– Valgus impacted 4-part dislocation fractures.
– Concomitant functionally relevant rotator cuff lesions.
– Concomitant glenoid fractures.
– Advanced omarthrosis.

### Anatomic fracture arthroplasty

The implantation of an anatomic fracture arthroplasty is exclusively indicated when a joint-preserving treatment is impossible in patients who verifiably unveil any lesions of the rotator cuff muscles and show good compliance. In the context of an anatomic fracture hemi-arthroplasty, the exact anatomic reconstruction as well as the osseous healing of the tuberosities are both pivotal to a reduction of the risk for premature erosions of the glenoid as well as to functioning rotator cuff muscles, essential to a sufficient function of the arthroplasty [26, 34, 35]. Large tuberosity fragments, anatomic reconstruction with good metaphyseal bone contact, stable primary fixation with vertical and horizontal sutures and/or wire cerclages as well as strict avoidance of bone cement in the fractured area represent indicators for a good osseous healing. To further optimize the functional outcome and to circumvent the risk of secondary glenoid defects, downgrading the function of the arthroplasty, the authors of this work recommend the simultaneous implantation of the glenoid replacement. Another option for reducing glenoid defects is the use of special materials, f.e., a pyrocarbon head. Fractures with a metaphyseal comminution zone and progressed destruction of the medial calcar segment were, however, found to hinder the successful implantation and function of an anatomic fracture arthroplasty. Multifragmentary fractures of the tuberosities and/or a reduced bone quality, moreover, impede the osseous consolidation [26]. Due to the facts that the surgical proceedings are technically demanding, that the sufficient functioning of the arthroplasty predominantly relies on the unpredictable osseous consolidation of the tuberosities [36] and that the preserving of the joint represents the predominant therapy objective in younger patients, the anatomical fracture prostheses have successively become less relevant in everyday clinical routine [15, 26].

### Reverse total shoulder arthroplasty

**Figure 3:**
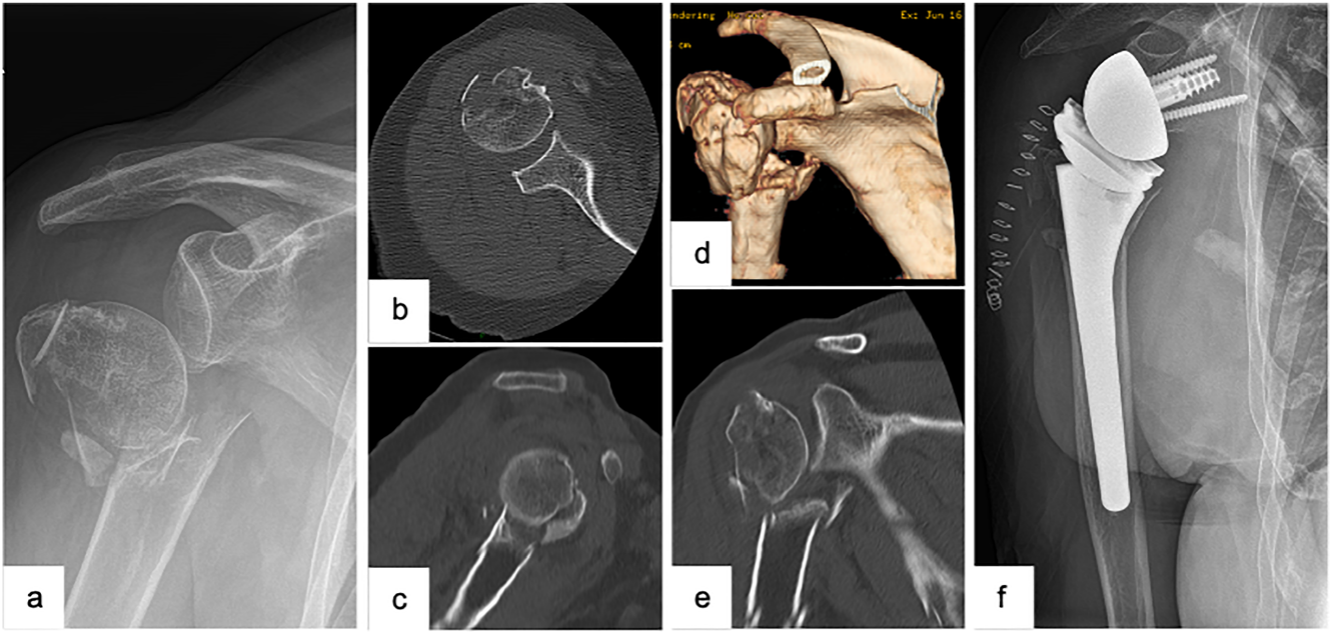
Female patient (83 y), right shoulder, varus distracted 4-part proximal humerus fracture (preoperative X-ray and (3D-) CT-scan: a – a.p., b – axial, c – sagittal, d – 3D-CT-reconstruction, e – coronal), implantation of a reverse total shoulder arthroplasty (postoperative X-ray: f – a.p.).

The implantation of a reverse total shoulder arthroplasty (Figure 3) represents an efficacious treatment option for those cases when a joint-preserving treatment or the implantation of an anatomic fracture prosthesis (e.g., preexisting lesions of the rotator cuff muscles, comminution zone in the area of the tuberosities, osteoporotic bone quality, concomitant fractures of the glenoid) is expected to result in poor clinical outcomes [15, 37]. In general, the two main advantages of a reverse total shoulder arthroplasty, are, on the one hand, its independence from the state of the rotator cuff muscles thanks to shifting the glenohumeral center of rotation and thus relying on the deltoid muscle to particularly initiate the shoulder abduction. On the other hand, the second advantage is its independence from the tuberosities and in particular from their anatomic reconstruction as well as their osseous healing. Clinical studies have recently reported considerable enhancement in activity and quality of life succeeding a successful implantation of a reverse shoulder arthroplasty [20, 37, 38].

Although the functioning of the reverse total shoulder arthroplasty does not depend on the reconstruction of the tuberosities, it has been divulged that reconstructed tuberosities are, nonetheless, advantageous to an ameliorated postoperative clinical outcome, in particular to an increased external rotator function [26, 37, 38]. As most activities of daily living require an external rotator function of approximately 60° [38], the reconstruction of the tuberosities should therefore always be performed. To hereby facilitate the latter, modern models of reverse arthroplasties have specific refixation possibilities incorporated in the prosthesis design. In order to further ameliorate the postoperative clinical range of motion, the baseplate should be positioned more inferiorly and the tendon of the supraspinatus should be partially or completely resected.

With regard to the most common complications of the implantation of a reverse shoulder arthroplasty with an overall complication rate up to 19.4 %, scapular notching accounts for one of the main complications apart from aseptic or septic loosening of the implant, instability or luxation of the arthroplasty, infections or periprosthetic fractures, particularly in patients with an osteoporotic bone quality [26, 38]. Notching implies a mechanical contact between the inlay of the prosthesis or the metaphysis of the humerus and the scapula neck, possibly resulting in an ablation of the inferior glenoid rim, consecutively leading to a secondary loosening of the arthroplasty or its secondary dislocation. However, the risk of notching could be reduced thanks to both optimized prosthesis designs and implantation techniques [26].

Postoperatively either succeeding the implantation of an anatomic or a reverse total shoulder arthroplasty, the follow-up treatment involves the intermittent immobilization of the respective shoulder in an abduction orthosis for in total 6 weeks. Except for the retroversion which is to be avoided, the range of motion is unlimited with at first actively assisted movements during physiotherapy and then with gradually more active ones. The latter are meant to train the correct positioning of the scapula and the centering of the humeral head.

With respect to the clinical outcome succeeding the implantation of a reverse total shoulder arthroplasty as a primary treatment of proximal humerus fractures, the latter qualified to be better in comparison to the clinical outcome following implantation of anatomic fracture prostheses [26]. Therefore, and in line with the aforementioned aspects, a clear preference has developed for the primary implantation of reverse shoulder arthroplasties in the context of arthroplasty treatment of proximal humerus fractures.

## Acromioclavicular joint dislocation

### Epidemiology and etiology

Acromioclavicular (AC) joint injuries are among the most common pathologies of the shoulder girdle, accounting for 4–12 % of all cases, making them very relevant in clinical trauma surgery and orthopedic practice [39]. The incidence can be reported as approximately 3–4 disorders per 100,000 population [40]. Regarding the gender distribution of AC joint injuries, the incidence is clearly on the side of male patients. In their study, Chillemi et al. [41] reported a male-to-female ratio of 8.5 to 1. Clinical retrospective studies of acute and chronic AC joint injuries by Jensen et al. [42, 43] have also reported a clear numerical predominance of the male sex. The average patient age of sufferers ranges from 20 to 40 years, and young and athletically active adults are most commonly affected [41, 44]. The typical mechanism of trauma is most commonly described as direct trauma to the posterosuperior shoulder with adducted arm, or less commonly, indirect trauma to the elevated arm. In the latter, the force is transmitted indirectly through the elevated acromion into the AC joint. Direct trauma usually occurs in a fall from a higher speed, and the affected person is often no longer able to support himself with his arm. Predisposed sports are especially cycling, motorcycling and alpine sports [45, 46]. In mountain sports, about 20 % of shoulder girdle injuries involve the AC joint [47]. In conclusion, it can be stated that instabilities of the AC joint can be considered as a relevant variable in the traumatological professional routine due to their frequency and the young patient clientele. Efficient clinical examination and diagnosis as well as appropriate therapy and follow-up treatment are relevant factors to be considered, especially with regard to the prevention of chronic instability.

### Examination findings and diagnosis

Clinical examination alone can provide a directional finding in AC joint injuries. Patients often enter the examination room with an arm in a painful posture. Even when looking at the unclothed patient, a malposition in the sense of a clavicle upright is sometimes conspicuous. Patients frequently report an isolated pain over the AC joint with likewise marked pressure dolence. In the clinical examination, one can not only check for vertical instability of the clavicle in the sense of a piano key phenomenon, but also assess the extent of horizontal instability by anterior-posterior translation of the clavicle relative to the acromion. Active range of motion of the affected shoulder is often significantly limited in the acute situation due to pain, especially during abduction and elevation of the arm. In more severe lesions, scapular depression due to caudal traction of the arm is also not uncommon. In chronic AC joint injuries, mobility in the shoulder joint often shows little restriction. Here, chronic pain and the development of scapular dyskinesia or SICK scapular syndrome are more likely to occur [48].

**Figure 4:**
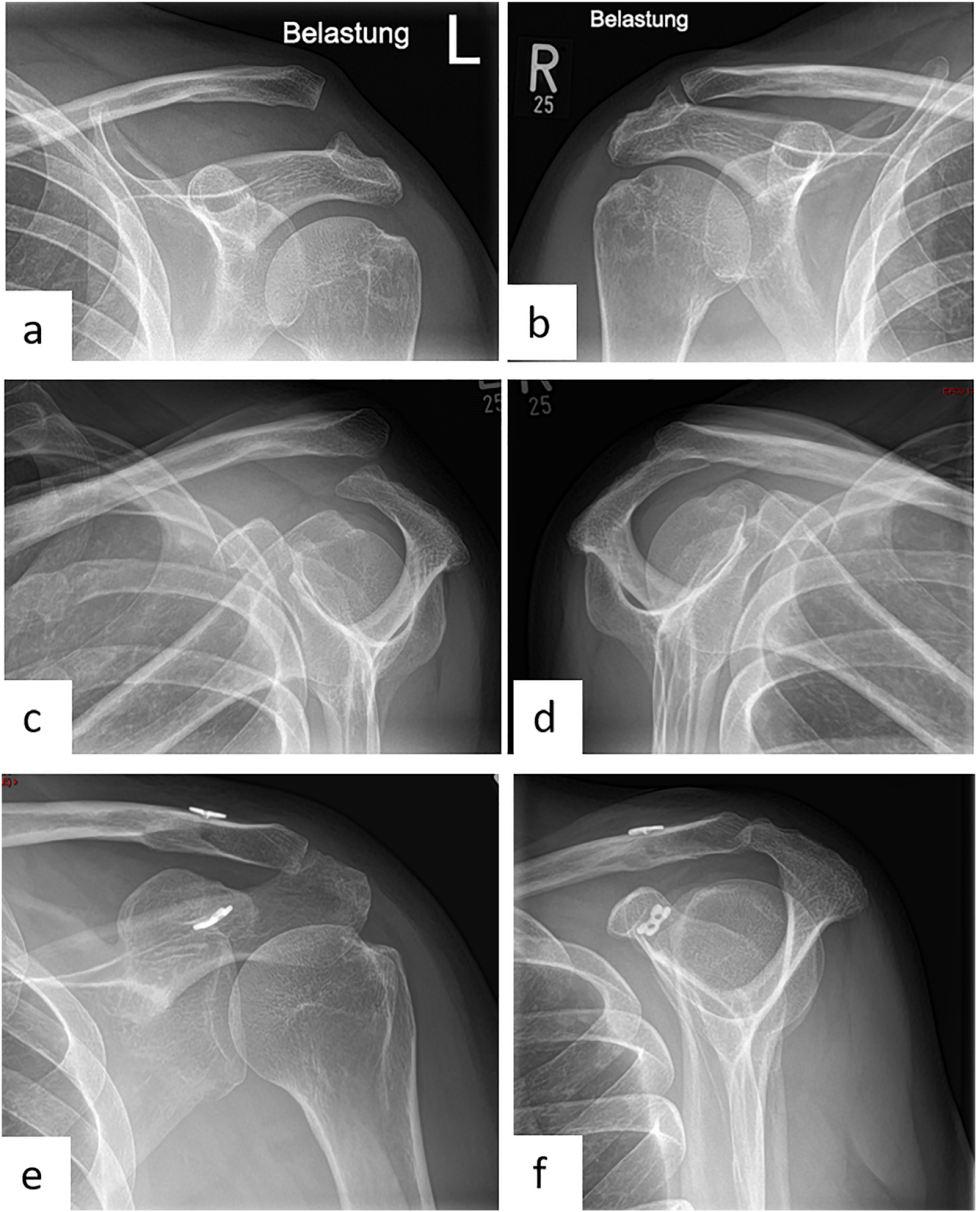
Female patient (50 y), left shoulder, acute acromioclavicular joint dislocation (Rockwood 5) (preoperative X-ray): a – Zanca stress radiographs with 10 kg weights left shoulder, b – Zanca stress radiographs with 10 kg weights right shoulder, c – Alexander radiograph left shoulder, d – Alexander radiograph right shoulder, arthroscopically supported vertical and horizontal stabilization with a TightRope, postoperative X-ray: e – Zanca, f – Outlet view.

The standard radiographic approach to suspected AC joint injury includes an axial radiograph of the affected shoulder. Here, posterior instability can be visualized. In addition, bilateral Alexander radiographs are performed to evaluate horizontal instability and Zanca stress radiographs with 10 kg weights are performed to show vertical instability in a lateral comparison (Figure 4) [45, 46, 49]. A panoramic image with imaging of the upper thoracic aperture looking at both AC joints should be avoided due to the high radiation exposure with the thyroid gland being particularly at risk. Furthermore, from a certain patient age, preoperative MRI diagnostics of the shoulder can be considered due to the increased incidence of glenohumeral concomitant pathologies [43]. However, this is not yet part of the diagnostic gold standard. CT diagnostics are reserved for special cases, such as a coracoid fracture.

### Classification

Injuries to the AC joint are classified into six different types according to Rockwood [53]. A strain or partial rupture of the acromioclavicular (AC) ligaments is evaluated as Rockwood I. However, the coracoclavicular ligaments (CC) are intact in this case. In Rockwood II, there is a complete tear of the AC ligamentous apparatus as well as a partial rupture of the CC ligaments. Here, radiographic diagnosis reveals a widening of the acromioclavicular joint space with an elevation of the lateral clavicle by less than half a shaft width. Finally, the Rockwood III type is considered to be a complete tear of the AC and CC ligaments. The radiograph in this case shows an upstanding clavicle by a complete shaft width. The question of non-operative or surgical treatment of Rockwood III injuries was very problematic and controversial for a long time. The ISAKOS (International Society of Arthroscopy, Knee Surgery & Orthopaedic Sports Medicine) has issued a more precise subclassification of Rockwood III injuries in this regard. The goal of this subclassification is to filter patients who will benefit from surgical therapy to avoid long-term complications such as scapula dysfunction due to an overlooked surgical indication. Depending on the presence of horizontal instability (anterior-posterior translation of the clavicle relative to the acromion), a type III A (without horizontal instability) and III B (with horizontal instability) injury is classified [54, 55]. Since an additional horizontal instability results in poorer clinical results, operative treatment is recommended [72]. If the injury is a Rockwood IV injury, the criteria for Rockwood III injury are evident. In addition, there is a lesion of the deltoideotrapezoidal fascia. Again, radiographic diagnosis reveals an elevation of the lateral clavicle with additional clinical and radiographic instability in terms of posterior dislocation. AC joint dislocations of the Rockwood V type are characterized by the criteria of a type IV injury. In addition, radiographs show elevation of the lateral clavicle by more than a full shaft width. Rockwood VI injuries are extremely rare and represent dislocation of the clavicle below the acromion or coracoid [53].

A basic distinction is made between acute and chronic AC joint injuries. If the traumatic event occurred 3 weeks or less ago, this is referred to an acute AC joint disruption. If the accident occurred 3 weeks or more ago, this is considered chronic AC joint instability. After 3 weeks the ligaments lose their potential of healing. This classification is very important, especially for surgical therapy and subsequent healing of the ligamentous apparatus [42, 45, 46, 50, 51]. Chronic AC joint instability can occur when the initial injury has been overlooked or left untreated. Chronic symptoms may also occur during the course of non-operative therapy or, less commonly, surgical treatment [52].

### Therapy

The definitive goals of therapy include regaining full functionality, pain-free motion, and preventing the development of chronic AC joint instability. Rockwood I and II injuries are treated non-operatively. In studies conducted for this purpose, good clinical results could be described with the non-operative therapy concept. In addition to pain-adapted analgesia, short-term immobilization in a Gilchrist bandage for a maximum of 1–2 weeks is recommended. This is followed by physiotherapy and range-of-motion exercises without weight-bearing. After 6 weeks, strength building is started, and healing should be complete after approximately 6–12 weeks [54, 56, 57]. However, long-term sequelae may also develop [48, 58]. Therapy for Rockwood type III injuries has been the subject of significant controversy in the previously published literature. According to study results, the development of scapula dyskinesia or SICK scapula syndrome occurs in more than 50 % of non-operatively treated type III injuries [48, 59]. Young and athletic patients in particular benefit from surgical therapy [56]. According to previous literature recommendations, Rockwood type III A injuries are treated non-operatively. However, in the presence of horizontal instability (Rockwood type III B), surgical treatment is indicated. For Rockwood type IV and V AC joint injuries, definitive surgical stabilization is recommended in agreement with previous literature [60]. However, the most recent literature is inconclusive regarding the definitive surgical approach to Rockwood type V injuries [82, 83].

Addressing not only the CC ligaments but also the AC ligaments is a prerequisite for optimal therapeutic success in order to achieve vertical and horizontal stabilization. In their study from 2015, Barth et al. [61] were able to determine that anatomic outcome directly correlates with functional outcome. It is shown that the stabilization of the CC ligaments alone is not sufficient and purposeful, because due to the non-exact anatomical reduction, a postoperative loss of correction can lead to a restriction of movement and persistent pain in the shoulder area in the long term.

Several studies published to date, including a recent paper by Dey Hazra et al. in 2023 [62], have shown that the postoperative clinical outcome of patients operated in the acute stage is better than that of those who underwent delayed surgery [61], [62], [63]. In their analysis, Dey Hazra et al. [62] compared clinical scores and the VAS-pain-scale of patients who received acute and chronic stage care: postoperative outcomes and functionality were significantly better in patients who received acute care. Other factors considered here included the time to return to work and the rate of postoperative sports activity. In the chronic AC joint injury group, 78 % of patients who underwent surgery were able to return to their previous occupation. In 15 %, the previously practiced profession had to be changed, and 6 % were unable to continue working in their profession. Seventy percent of the operated patients were able to resume their sport after some time. In 27 % it was necessary to change to another sport and in 3 % no sporting activity could be performed at all. In contrast, 94 % of patients who underwent surgery for acute AC joint dislocation were able to return to their previous occupation. In 79 %, the previously performed sports activity could be resumed and practiced. Dey Hazra et al. [62] have also reported a revision rate after surgical stabilization of chronic AC joint injuries of 12 %. When acute AC joint dislocations were treated, the revision rate was 9 %. In most cases, this has been due to material irritation of the buttons, although the clavicular button seems to be significantly more predisposed. For the clinical traumatological daily routine a clear relevance is shown regarding the timing of surgery: the goal should be to diagnose AC joint injuries in the acute stage and, if necessary, to treat them surgically in a timely manner. In this way, the development of chronic processes is minimized, which ultimately leads to an improvement of the clinical outcome for the affected patients.

The general question of the optimal surgical treatment of AC joint injuries has been controversial for a long time. In the literature to date, more than 160 different surgical techniques have been described. Basically, a distinction is made between an open and an arthroscopic procedure. Among others, Stein et al. [64], Arirachakaran et al. [65], and Lloyd et al. [66] reported significantly better postoperative functional results of minimally invasive arthroscopic treatment. In the open procedure, only osteosynthetic treatment using a hook plate plays a role in today’s clinical practice. The advantage of this procedure is that stabilization is easy to implement in terms of surgical technique. However, the use of a hook plate has many disadvantages and postoperative complications: a follow-up operation to remove the material after 3 months is obligatory. In addition, local irritation of the plate may result in acromion osteolysis and fracture, subacromial impingement, and rotator cuff damage, among others. Postoperative loss of correction, especially in the horizontal plane, is also not uncommon [45, 46, 50, 67, 68]. A survey published by Balke et al. [69] showed that shoulder specialists clearly prefer the use of arthroscopically supported “pulley” systems over the hook plate, especially since glenohumeral concomitant pathologies can be identified and addressed. In recent years, various double-button implants to implement vertical stabilization and additive transacromial suture cerclage to restore horizontal stability have become particularly popular [49, 70]. Previous literature has reported excellent results with the arthroscopically assisted technique, although it is essential to pay attention to the additional horizontal stabilization [64, 71, 72]. Particular advantages of arthroscopically assisted therapy are, on the one hand, a minimally invasive and tissue-conserving surgical technique as well as a significant reduction in postoperative correction loss in terms of posterior translation due to the additive horizontal cerclage [50]. Glenohumeral concomitant pathologies can be identified and addressed if necessary, which can be considered a decisive advantage, among others [43]. Furthermore, high patient acceptance has been described, sometimes due to satisfactory functional and cosmetic results [50, 64]. There is no mandatory material removal. In a study by Jensen et al. [73] on arthroscopic AC joint stabilizations, postoperative satisfaction of the operated patients was shown in 97 % of the cases. In particular, the good functional outcome was emphasized, as all operated patients did not retain any mobility limitations. Postoperative migration of the clavicular or coracoid buttons was not observed in any patient in this study. However, disadvantages of the arthroscopic procedure are a higher technical effort for the surgeon and a possible material irritation, especially of the clavicular button. The development of iatrogenic clavicle or coracoid fractures during placement of the drill is also a rare but possible complication [45]. To prevent the fractures, care should be taken to use drills with a narrow diameter (2.4 mm). Previously published biomechanical studies have reported a significant effect of the number of drill holes and the drill hole diameter on the stability of the coracoid and clavicle [74, 75]. A positive trend towards minimization of the drill hole diameter could be achieved by the newer Endobutton techniques, because here not the implant itself but only the suture material is guided through the drill channel.

### Therapy of acute AC joint injuries

The senior authors institution suggests that acute AC joint dislocations are treated arthroscopically using the Endobutton technique (Figure 4). Anesthesia is performed under general anesthesia. The patient is positioned in beach chair position with head rest, and a pneumatic arm holder is mounted for controlled movement of the arm. A sterile integrated image transducer for visualization of the burr channels is positioned. The three standard portals (posterior, anteroinferior, anterolateral) are set for arthroscopic access. First, a diagnostic reflection of the shoulder joint is performed via the posterior portal. Possible concomitant pathologies can be detected here and, if necessary, directly corrected. After the diagnostic arthroscopy, a lateral transtendinous arthroscopic approach is made, through which optical imaging is now performed. From the lateral view, arthroscopic visualization of the coracoid arch can now be performed. Via the anterioinferior working portal, the subcoracoidal preparation is now performed in the first step. Next, an approximately 2–3 cm longitudinal skin incision is made above the lateral clavicle to prepare it as well. Anatomical reduction is achieved by applying caudoventral pressure to the AC joint with additional cranialization of the arm via the arm holder. A slight overcorrection is recommended in accordance with the study to counteract postoperative loss of correction. Reduction is held by a k-wire. To perform the transclavicular-transcoracoidal drilling for vertical stabilization, the insertion handle is placed below the coracoid base. The drill is performed under visual arthroscopic and image intensifier-assisted radiological control with an entry point between the attachments of the two CC ligaments and an exit point at the base of the coracoid. In the next step, a shuttle suture is now used to place the Endobutton system with double button implant (e.g., DogBone with Tiger and FiberTape, Arthrex, Naples, FL, USA). The buttons come to rest inferior to the coracoid and over the clavicle. The suture of the clavicular button is left undetached. In the next step, minimally invasive horizontal stabilization is performed using suture cerclage. Via a lateral stab incision, transacromial drilling is performed with subsequent overdrilling by a cannulated drill (2.7 mm) under radiological control. Finally, one leg of each tape is passed laterally through the drill channel with the aid of a shuttle wire and then returned. Finally, the tapes are tightly knotted together under lateral pressure on the acromion. The result is seen in vertical and horizontal stabilization (Figure 4). The joint space of the AC joint should be reduced again to a normal value between 1 and 6 mm. Finally, closure of the deltotrapezoidal fascia and suturing of the wound is performed. A sterile wound dressing is applied and the patient is removed from the bed.

### Therapy of chronic AC joint injuries

**Figure 5:**
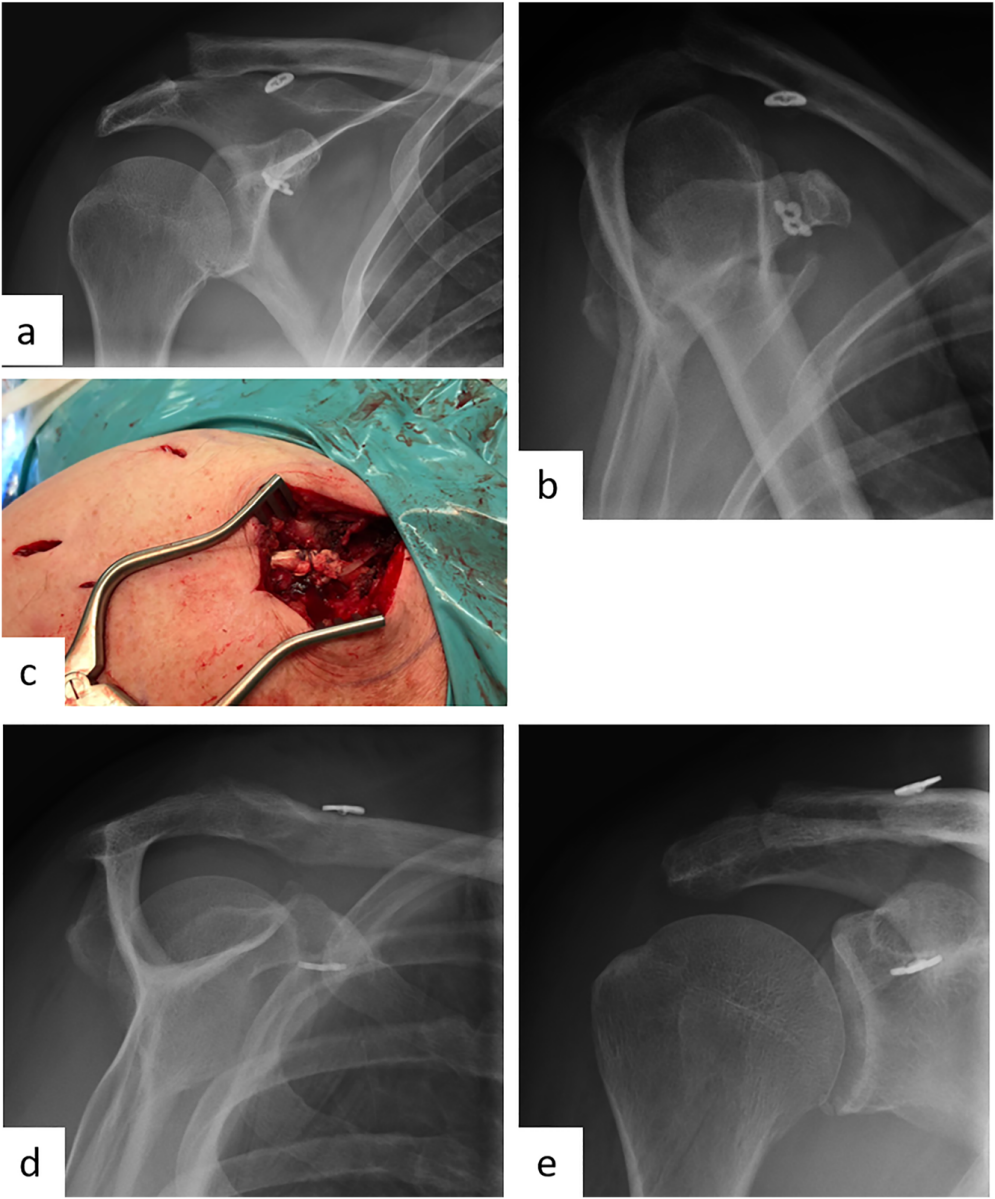
Male patient (55 y), right shoulder, chronic acromioclavicular joint instability with loss of reduction after surgical displacement of an acute Rockwood 3B injury 3 months ago (preoperative X-ray): a – Zanca stress radiograph with 10 kg weight, b – Alexander radiograph, c – arthroscopically supported vertical and horizontal stabilization with a TightRope and an additional autologous Hamstring-tendon augmentation, postoperative X-ray: d – Outlet view, e – a.p.

If the trauma leading to the injury occurred more than 3 weeks ago, it is referred to as chronic AC joint instability. Maier et al. [76] described in their study that with increasing time after the trauma a progressive decrease of the healing potential of the ligamentous structures can be seen. This can be seen as a cause of increasing fibroblast-like cell proliferation as well as tissue remodeling. Overall, the formation of a scar volume necessary for stabilization is no longer guaranteed after a longer period of time, which is why the surgical therapy of acute and chronic AC joint injuries differs. Several surgical techniques have also been described in recent years for the treatment of chronic AC joint instability, including in particular extraanatomical procedures and biological augmentation. Biologic augmentation is the use of autologous tendons to stabilize the AC joint. The use of hamstring tendons (*M. gracilis*, *M. semitendinosus*) is most common in this context. Several studies have described significantly better clinical and radiological results after a biologic augmentation compared with the other procedures. In particular, the use of hamstring tendons in combination with an Endobutton system achieved excellent clinical results [77], [78], [79]. The ESA consensus statement also clearly recommends the use of autologous tendon material in chronic cases [84]. In many countries, it is now also possible to use allografts, which have become increasingly established as the surgical standard. In German-speaking European countries, however, their use is limited, as there are legal regulations that serve the safety of the patient and therefore make the use of these tissues more difficult in some cases [85]. The senior authors institution suggests that arthroscopically assisted AC joint stabilization is performed using the Endobutton and loop tendoplasty (Figure 5). For this purpose, a diagnostic arthroscopy and vertical stabilization with double-button Endobutton (e.g., DogBone, Arthrex) are performed first, as already described for the treatment of acute AC joint injuries. In the next step, the semitendinosus tendon is then harvested from the pes anserinus of the sterilely washed and covered ipsilateral leg. After preparing the tendon to a diameter of approximately 4 mm and a minimum length of 24 cm, a soft tissue passage is prepared posteromedial to the clavicle to subacromial medial to the coracoid. A shuttle suture is placed. Another soft tissue passage is prepared ventrolateral to the clavicle, over which the shuttle suture is again passed and carried out cranially. In the following step, the semitendinosus tendon is tightened over the shuttle suture and looped around the coracoid. Anterior-posterior stabilization is achieved by the ventral and dorsal passage of the tendon in relation to the clavicle. The two ends of the tendon are now twisted together and sutured, leaving the dorsomedial end of the tendon long. Finally, transacromial drilling is performed (see therapy of Acute AC joint injuries). The tendon end that is left long is pulled transacromially via a shuttle suture placed in the drill channel and discharged subcutaneously, where suturing of the two tendon ends finally takes place. Finally, the wound is closed in layers.

The follow-up treatment of acute and chronic AC joint injuries is as follows in the authors’ procedure: the shoulder-arm-sling should be worn for 6 weeks, especially to avoid traction on the shoulder by drooping of the affected arm. During the first 2 weeks, physiotherapeutic exercises are performed with a maximum abduction and anteversion of 60° and an internal rotation of 80°. From the third week, abduction and anteversion can then be increased to 90° each. From the third month, the increasing load should be trained. Finally, after reaching the seventh week, there is no more limitation of movement. Participation in contact sports should not be resumed until after approximately 9 months.

### Concomitant glenohumeral pathologies

The incidence of glenohumeral concomitant pathologies that may present during surgical arthroscopic treatment of AC joint injuries has been reported in the literature to date to range from 15 to 53 % [42, 50, 80]. Jensen et al. [43] published a retrospective study in 2017 (data from Hanover and Vail), describing 376 patient cases who underwent arthroscopic AC joint stabilization for Rockwood type III and V injuries from January 2007 to December 2015. Arthroscopically detected concomitant glenohumeral pathology was described in 53 % of the cases described. In most cases, the anterosuperior rotator cuff and the biceps tendon complex were affected. Overall, one or more pathologies were present in 12 % of the patient cases described, necessitating additional reconstructive therapy. Most frequently reconstructed were lesions of the biceps tendon with 9.6 %, followed by pathologies of the rotator cuff with a share of 5.1 %. The supraspinatus and subscapularis tendons were particularly affected. Sometimes for this reason, arthroscopic AC joint stabilization with prior diagnostic arthroscopy is much preferred to the open approach. Arrigoni et al. [80] and Jensen et al. [43] found a significant increase in glenohumeral concomitant pathologies and their need for reconstruction with increasing age. Patient age was even considered to be the most significant influencing factor. In this regard, it is a justified consideration that the indication for preoperative performance of MRI diagnostics of the shoulder should be established in clinical routine from a certain age (for example, from the age of 50) for more efficient surgical planning and patient preparation. Overall, a high degree of agreement between MRI morphologic and arthroscopic diagnostics has been reported with regard to the visualization and detection of concomitant glenohumeral injuries [81]. Jensen et al. [43] also report an increased incidence of glenohumeral concomitant pathologies in the context of chronic AC joint instability with a proportion of 61 %, whereby the difference here is even significant in comparison to the acute injury. The need for additional reconstruction of the concomitant pathology was also shown to be significantly higher in chronic injuries. With a proportion of 60 %, concomitant pathologies were described more frequently in patients with a Rockwood type V injury, compared with 47 % in Rockwood type III injuries. However, the severity of the injury did not significantly influence the need for reconstructive therapy.

## Conclusions

AC joint injuries are a common pathology to the shoulder girdle. It mainly affects young and athletically active men. The therapy depends on the age of the injury (acute or chronic) and the Rockwood type. In recent years, arthroscopically supported Endobutton systems have become established for surgical treatment. In this way, additional horizontal stabilization can be ensured, which is decisive for the postoperative functional result. In addition, glenohumeral concomitant pathologies can be detected and addressed if necessary. Due to a decreasing healing potential of the ligaments in chronic AC joint instabilities, an additional reinforcement with an autologous tendon (Hamstring tendons) is performed.

## Supplementary Material

Supplementary Material
