# Supplementary material for: Proximal humerus fracture and acromioclavicular joint dislocation
Source: Innov Surg Sci. 2024 Apr 11;9(2):67–82. doi: 10.1515/iss-2023-0049 (PMC11294519; doi:10.1515/iss-2023-0049)
Supplement: Supplementary file 1 — Supplementary Material [file j_iss-2023-0049_suppl_001.pdf]

Reviewer Assessment

F. Reeh et al.: Proximal Humerus Fracture and Acromioclavicular Joint Dislocation

Reviewers' Comments to Original Submission

Reviewer 1: anonymous

Date received: 01-Oct-2023
Reviewer recommendation: **Return to author for minor modifications**
Reviewer overall scoring: **High**

Comments to author:
Thanks This is an interesting review investigating the current evidence on the proximal humeral fracture and AC-Joint dislocations. The authors should be commended for their effort in completing it. However, I do believe there are minor revisions needed. The manuscript should shortened and each paragraph should reflect a clear message.

All in all, this review is a well written and should be published after below mentioned changes.

Title
Ok.

Abstract

missing

Background

Line 23-26 Please delete

Line 29 : Please change triple to three times more etc.

Line 47-54: Please delete.

Classification :
Please shorten and focus on the main classifications - Neer, Cadman and Resch. Maybe with a short table. Do not draw conclusions or therapeutic strategies in this segment. Please delete 32-40.

Line 52:
Please change Conservatively, non surgically etc throughout the manuscript to non-operatively

Line 21: Please change Gillchrist to shoulder-arm-sling

Surgical treatment:

Line 49-57:
Maybe add some authors choose the therapeutic strategies independent of the age

Open reduction and internal fixation with locking plate osteosynthesis

Please change to Locking plate fixation throughout the manuscript

Please change endprosthetic to arthroplasty throughout the manuscript

Anatomic fracture arthroplasty

Please remove young - the main indication for a anatomic arthroplasty is a intact cuff !

.....However, the risk of notching could be reduced thanks to both optimized prosthesis designs (e.g. reduction of the glenohumeral inclination from 155° to a more varus one of 135°, further lateralization) and implantation.....

Please delete the options or further explain further.

Acromioclavicular Joint dislocation

Classification
Line 58:
Please do not start with acute and chronic. Start with the Rockwood Classification , ISAKOS Consensus statement with 3B etc. Then if needed explain the ESA consensus statement regarding the need of a tendon autograft for chronic cases after 21 days. If so, please explain why this is a Europe based problem (Allograft etc.)

Therapy
Line 3: Please add recent literature is inconclusive regarding the definitive surgical approach in Rockwood type 5 cases. Windhamre/Ekelund study , Canadian register study etc.

Therapy acute / chronic :
Please add the senior authors institution suggests etc.

Concomitant pathologies :
Please reduce.

Conclusions :
Please change Tight Rope to Endobutton or suture button systems,.

Reviewer 2: anonymous

Date received: 08-Oct-2023
Reviewer recommendation: **Return to author for minor modifications**
Reviewer overall scoring: **High**

Comments to author:
Thank you for your submission. This is an interesting review regarding proximal humeral fractures and injuries to the ac joint.
I have few comments to improve the paper:
p1, line 24: most common fracture
p1, line 38-40: you could also argue that some experts choose conservative treatment even for some of the displaced fractures
p1, line 46: ideal treatment of displaced proximal humerus
p1, line 60/1: individual demands
p2, line 24-27: this section about x-ray diagnostic could be confusing for the reader, please clarify
p2, line 59: "calotte" is not a typically used term in this context
p4, line 33: orthesis
p7, line 16: please clarify you are talking about a hemi arthroplasty when talking about erosion a a glenoid
p7, line 29: you could mention the possibility of using diffenrent materials f.e. a pyrocarbon head for less glenoid erosion

p11, line 6 et sqq.: please clarify on the indirect stabilization of the cc-ligaments/ac joint capsule
p11, line 60: replace tightrope with "pulley", as tightrope is a brand name
p13, line 36: tendoplasty is not a commonly used term in this context as is refers to the reparative surgery of the tendon, not the biological augmentation described here
p14, line 4: orthesis
and what kind?

Otherwise, this is a good contribution to our literature.

Authors’ Response to Reviewer Comments

Date received: 29-Nov-2023

Reviewer 1:

Abstract:

Missing

- A short introductory abstract has been written and inserted. In our opinion, a more comprehensive description in the abstract should be omitted, as this is provided in the introductions to the two chapters on proximal humerus fractures and acromioclavicular joint injuries.

Background:

Line 23-26 Please delete

- Lines 23 to 26 have been deleted

Line 29 : Please change triple to three times more etc.

- The text has been changed accordingly

Line 47-54: Please delete.

- Lines 47 to 54 have been deleted

Classification:

Please shorten and focus on the main classifications - Neer, Cadman and Resch. Maybe with a short table. Do not draw conclusions or therapeutic strategies in this segment. Please delete 32-40.

- The section on classifications has been significantly shortened.
- Therapeutic strategies and consequences mentioned by us have been deleted.
- Lines 32 to 40 have also been deleted.
- We do not consider a table appropriate at this point and have therefore decided to formulate it in the text.

Line 52: Please change Conservatively, non surgically etc throughout the manuscript to non-operatively

- This change has been made consistently throughout the text.

Line 21: Please change Gillchrist to shoulder-arm-sling

- It was changed accordingly.

Surgical treatment:

Line 49-57: Maybe add some authors choose the therapeutic strategies independent of the age

- That is true. This aspect has now been added to the text.

Open reduction and internal fixation with locking plate osteosynthesis

Please change to Locking plate fixation throughout the manuscript

- The entire text has been changed accordingly.

Please change endprosthetic to arthroplasty throughout the manuscript

- The entire text has been changed accordingly.

Anatomic fracture arthroplasty

Please remove young - the main indication for a anatomic arthroplasty is a intact cuff!

- „young“ has been removed in the text.

.....However, the risk of notching could be reduced thanks to both optimized prosthesis designs (e.g. reduction of the glenohumeral inclination from 155° to a more varus one of 135°, further lateralization) and implantation.....

Please delete the options or further explain further.

- The options have been deleted.

Acromioclavicular Joint dislocation

Classification:

Line 58: Please do not start with acute and chronic. Start with the Rockwood Classification, ISAKOS Consensus statement with 3B etc. Then if needed explain the ESA consensus statement regarding the need of a tendon autograft for chronic cases after 21 days. If so, please explain why this is a Europe based problem (Allograft etc.)

- The explanation of the Rockwood classifications and ISAKOS Consensus statement was placed accordingly at the beginning, whereupon the distinction between acute and chronic is made.

- The ESA consensus statement and the problem of the use of allograft have been added to the text. However, it was not mentioned in the Classifications subsection, but in the section on the treatment of chronic ACJ injuries, as we believe it fits better here in terms of content.

Therapy:

Line 3: Please add recent literature is inconclusive regarding the definitive surgical approach in Rockwood type 5 cases. Windhamre/Ekelund study, Canadian register study etc.

- „However, the most recent literature is inconclusive regarding the definitive surgical approach to Rockwood type V injuries." Has been added.

- Windhamre/Ekelund study and Canadian register study have been added.

Therapy acute / chronic:

Please add the senior authors institution suggests etc.

- It has been added accordingly in the text.

Concomitant pathologies:

- The section on concomitant pathologies has been significantly shortened. In our view, further shortening is not sensible due to the importance of this aspect.

Conclusions:

Please change Tight Rope to Endobutton or suture button systems.

- It was changed accordingly.

Reviewer 2:

The p1, line 24: most common fracture

- It was changed accordingly.

p1, line 38-40: you could also argue that some experts choose conservative treatment even for some of the displaced fractures

- This aspect has been added to the text accordingly.

p1, line 46: ideal treatment of displaced proximal humerus

- It was changed accordingly.

p1, line 60/1: individual demands

- It was changed accordingly.

p2, line 24-27: this section about x-ray diagnostic could be confusing for the reader, please clarify

- This section has now been slightly reworded and should be clearer and easier for the reader to understand.

p2, line 59: "calotte" is not a typically used term in this context

- „Calotte“ has been deleted.

p4, line 33: orthesis

- It was changed accordingly.

p7, line 16: please clarify you are talking about a hemi arthroplasty when talking about erosion a glenoid

- It has been added to the text that this is the hemi arthroplasty.

p7, line 29: you could mention the possibility of using diffenrent materials f.e. a pyrocarbon head for less glenoid erosion

- „Another option for reducing glenoid defects is the use of special materials, f.e. a pyrocarbon head“ has been added to the text.

p11, line 60: replace tightrope with "pulley", as tightrope is a brand name

- It was changed accordingly.

p13, line 36: tendoplasty is not a commonly used term in this context as is refers to the reparative surgery of the tendon, not the biological augmentation described here

- Tendoplasty was replaced by biologic augmentation.

p14, line 4: orthesis and what kind?

- shoulder-arm-sling-orthesis

Comments by the Editorial Office to the Editor-in-Chief Decision

All reviewer comments were addressed adequately and the manuscript should be published in its present stage.
